# Effect of Electroacupuncture on Cell Apoptosis and ERK Signal Pathway in the Hippocampus of Adult Rats with Cerebral Ischemia-Reperfusion

**DOI:** 10.1155/2015/414965

**Published:** 2015-11-08

**Authors:** Chunxiao Wu, Jiao Wang, Chun Li, Guoping Zhou, Xiuhong Xu, Xin Zhang, Xiao Lan

**Affiliations:** ^1^School of Traditional Chinese Medicine, Southern Medical University, Guangzhou, Guangdong Province 510515, China; ^2^College of Acupuncture, Moxibustion, and Tuina, Hunan University of Chinese Medicine, Changsha, Hunan 410007, China

## Abstract

*Background*. EA therapy is a traditional therapeutic approach for alleviation of cerebral I/R-induced brain injury. We investigated the effect of EA on MCAO rat model to examine the mechanism of apoptosis in the rat hippocampus.* Methods*. 200 male Sprague-Dawley rats were randomly divided into sham, I/R, EA, ERK inhibitor (PD), and ERK inhibitor+EA (PD+EA) groups. Each group was subdivided into 5 groups according to different time points. Locomotor behaviors were evaluated using neurological scales and morphological examination was performed using HE staining. Apoptosis index of neural cells in local infarcted area was measured by TUNEL and p-ERK expression was detected using immunohistochemistry technique and western blot analysis.* Results*. Neurological deficit scores and neural apoptosis in the EA group were lower than I/R group at the same time points, respectively. At different time points, p-ERK level was increased in the ischemic hippocampal CA1 in the EA group as compared to I/R group; the increased level was increased most at 1 day, 3 days, and 1 week (*p* < 0.01).* Conclusion*. EA alleviates neurological deficit, reduces apoptosis index, and simultaneously upregulates the expression of p-ERK signal pathway in rats subjected to I/R injury.

## 1. Introduction

Ischemic stroke is associated with high morbidity and mortality, and it is one of the most severe diseases affecting human health and causing death worldwide [1]. Ischemic/reperfusion results in hypoxia and apoptosis and damages nerve cells in the brain. Acupuncture, as a type of traditional therapy, has been widely used for improving cerebral vascular diseases and alleviating neurological disorder [2]. It has achieved good outcomes in treatment and rehabilitation of ischemic stroke [3, 4]. It has been shown that electroacupuncture (EA) enhanced proliferation and differentiation of neural stem cells, which is beneficial for recovery of damaged brain cells and for improvement of neurological deficit scores in rats subjected to cerebral ischemic/reperfusion (I/R) injury [5, 6]. Moreover, “Chize (LU5),” “Hegu (LI4),” “Zusanli (ST36),” and “Sanyinjiao (SP6)” are common acupoints for EA treatment for ischemic stroke [7], which can improve neurological deficit symptoms in ischemic stroke patients. However, the mechanisms underlying EA treatment for improving neural cells damage are complicated, and current researches about the mechanisms are still unclear. In the present study, we examined the mechanisms underlying EA by using it as an intervention approach.

The blood vessels after cerebral I/R can recanalize themselves. While delayed neuronal death induced after recanalization was mainly mediated through cell apoptosis [8], it has also been demonstrated that mechanisms underlying cerebral I/R injury were mainly associated with inflammation and cell apoptosis [9–11]. Mitogen-activated protein kinases (MAPK) cascades play a critical role in signal transduction of cellular apoptosis, including expression of extracellular signal-regulated kinases (ERK), c-Jun N-terminal protein kinases (JNK), and p38 mitogen-activated protein kinases [12]. Every signal pathway functions independently or interacts with other pathways, which contributes to studying mechanisms underlying cerebral I/R injury. Owing to the complexity of the signal transduction pathway mechanism, we examined the roles of ERK signal pathway in cerebral I/R injury using ERK signaling pathway as an indicator for observation.

ERK signal pathway is a main component of MAPK signal family, which is mainly associated with cell differentiation, cell proliferation, and apoptosis [13]. It has been shown that ERK1/2 is highly expressed in the ischemic brain, particularly in the hippocampus [14, 15]. While the activation of ERK is found to be associated with antiapoptotic effect [16, 17], various studies have shown that ERK activation could also induce apoptosis [18], whereas inhibition of ERK signaling pathway exerts neuroprotective effect [19]. Currently, there is controversy about the protective or apoptotic effect of ERK signaling pathway on the neural cells in response to the cerebral I/R injury. In the present study, we examined the expression of ERK signaling pathway in the CA1 area of the hippocampus and its role in regulating mechanisms of apoptosis using EA. Additionally, detailed effect of ERK on cerebral I/R injury was studied using ERK inhibitor PD98095 to confirm the role of ERK signaling pathway.

In the present study, apoptosis index and p-ERK protein level in neural cells were measured at 2 h, 6 h, 1 day, 3 days, and 1 week. Meanwhile, EA intervention once daily was conducted at 3 d and 1 week, which was adapted to clinical treatment approach. The once-daily intervention changed conventional experimental design for time points [20]. We studied the effects of different time points and various interventions on the expression of ERK signaling pathway in order to determine the best time window for EA treatment.

Based on the aforementioned studies, EA therapy was performed using a combination of acupoints of “Chize (LU5),” “Hegu (LI4),” “Zusanli (ST36),” and “Sanyinjiao (SP6).” In order to verify the hypothesis of “acupuncture stimulation-initiation of ERK signaling regulation-resistance to cell apoptosis” in cerebral I/R injury, locomotor behavior was assessed using neurological deficit scales, morphological examination was performed by hematoxylin-eosin (HE) staining, apoptosis was detected in the neural cells in the local lesion using terminal deoxynucleotidyl transferase dUTP nick end labeling (TUNEL) method, and p-ERK protein level was measured in the injured hippocampus using immunohistochemistry and western blot analysis.

## 2. Materials and Methods

### 2.1. Experimental Materials

#### 2.1.1. Animals

Adult healthy male Sprague-Dawley rats (*n* = 200), weighing 250–300 g, were obtained from the Center for Laboratory Animal, Southern Medical University (license number SCXK (Yue) 2011-0015). Animals were fed with standard commercial rodents chow, with free access to water ad libitum. The rats were acclimated in animal house for one week prior to the experiment; room temperature (RT) was controlled between 20 and 22°C and relative humidity at 65–70%. Prior to the experiment, the rats were housed in a quiet environment for 24 h and were fasted for 12 h prior to surgery but were given free access to water.

All the animal treatments were strictly in accordance with the International Ethical Guidelines and the National Institutes of Health Guide concerning the Care and Use of Laboratory Animals, and the experiments were approved by the Institutional Animal Care and Use Committee of Southern Medical University.

#### 2.1.2. Equipment and Reagents

One-inch disposable sterile acupuncture needles (diameter: 0.3 mm × 25 mm) (Suzhou Medical Instrument Factory, Suzhou, China), KWD-808I versatile pulse electronic acupuncture electrotherapy machine (Suzhou Universal Acupuncture Medical Devices Co., Ltd., Suzhou, China), and CJ-2F medical clean bench (Suzhou City FengShi Animals Equipment Co., Suzhou, China) were used for the study. For the study, DSC-RX100M2 digital camera (Sony Company, Japan), Bx-70 microscopy imaging system (Olympus Company, Suzhou, China), XS-200 binocular microscopy (Jiangnan Optics Co., Ltd., Nanjing, China), chloral hydrate (Aladdin Reagent Shanghai Co., Ltd., Shanghai, China), dimethyl sulfoxide (DMSO) (Sigma, Co., St. Louis, MO, USA), ERK inhibitor PD98059 (Sigma, Co., St. Louis, MO, USA), immunohistochemistry kit (Beijing Zhongshan Golden Bridge Biotechnology Co., Ltd., Beijing, China), and TUNEL test kit (KGI Nanjing Biological Technology Development Co., Ltd., Nanjing, China) were used.

### 2.2. Methods

#### 2.2.1. Grouping

Random number table method was used for experimental grouping: sham operation (sham) group, I/R group, EA group, ERK inhibitor (PD) group, and EKR inhibitor+EA (PD+EA) group (*n* = 40 rats per group). Each group was subdivided into 5 subgroups: 2 h group, 6 h group, 1 day group, 3 days group, and 1 week group (*n* = 8 rats per group). Following the establishment of I/R injury in rat model, different interventions were given after 1.5 h of establishing I/R model: rats in the sham and I/R groups were restrained on rat board in a supine position for 20 min without other treatments; rats in the EA group were restrained on rat board and EA stimulation of a combination of acupoints of “LU5,” “LI4,” “ST36,” and “SP6” was given; needle retention time was 20 minutes (min); in PD group, lateral ventricular injection of PD98059 was given to rats, 20 min after the injection. After 1.5 h of establishing I/R in rat model, the rats were restrained in a supine position for 20 min without any other further treatments; in PD+EA group, the rats were treated the same as those in the PD group, but they were provided with EA stimulation of a combination of acupoints of “LU5,” “LI4,” “ST36,” and “SP6”; needle retention time was 20 min.

#### 2.2.2. A Rat Model of Cerebral Ischemia/Reperfusion

Middle cerebral artery occlusion (MCAO) was used for establishing I/R model using Longa's modified method [21]. The rats were fasted for 12 h prior to surgery but were given free access to water. The detailed operation was made as follows: rats were anesthetized with intraperitoneal injection of 10% chloral hydrate (0.35 mL/100 g); 2 cm longitudinal cut was made on the skin at 0.3 mm of the left cervical anterior midline. The left common carotid artery (CCA), external carotid artery (ECA), internal carotid artery (ICA), and vagus nerve were exposed. Thread was inserted through CCA, ICA, and ECA, but without ligation; ligation of the CCA and ECA was made proximal to the heart; ligation of ICA was temporarily closed by a microvascular clamp; a small cut at 3 mm at the branch of ICA and ECA was made. A monofilament nylon suture was inserted (18.5–19.5 mm) from the CCA into the ICA until it was blocked by the clamp; the clamp was loosened and the suture was rapidly inserted further into the ICA. The angle of insertion of the suture was adjusted (inclining to the right approximately 15 degrees), and the CCA was gently pulled to make the suture enter the brain. When the depth of the suture was approximately 18 ± 0.5 mm (calculated from the branch of the blood vessel) and slight resistance is observed during insertion, slight resistance ensures that the suture, inserted in the MCA and MCAO, is successfully established. The ICA was tightly ligated and the wound was sutured. Reperfusion was made 30 min after MCAO: the suture was gently pulled back to the CCA, and the rest of the suture was cut off. Animals in the sham group were subjected to the same surgical procedures, except that they were exposed only to MCA, but without occlusion with insertion of nylon suture into the MCA. Rats were kept in separate cages for observation after the wound was sutured and were sterilized with povidone-iodine tampons. Once life signs of rats were stable, evaluation of the MCAO model was made according to Longa 5 grading scores method: 1–3 scores indicate successful establishment of MCAO model. Rats were housed in a separate single cage at RT at 20°C, with free access to food and water (water was administered by a dripping tube). After dissecting the brains of the MCAO rats, infarction lesion was located at the temporal lobe, which was consistent with the distribution of the cerebral blood supplied by the MCAO, suggesting that MCAO model was successfully established. If obvious subarachnoid bleeding was observed during dissection of the brain in a rat, the rat was excluded from the experiment.

#### 2.2.3. Electroacupuncture Stimulation Method

EA stimulation was conducted 1.5 h after MCAO. Selection of acupoints and EA stimulation were made according to “experimental acupuncture” edited by Li [22].

Selection of acupoints is as follows: as for LU5, in the depression of outer end of the transverse cubital crease, an acupuncture needle was inserted perpendicularly to a depth of 3 mm. As for LI4, located between 1st metacarpal bone and 2nd metacarpal bone, an acupuncture needle was inserted perpendicularly to a depth of 1 mm; as for ST36, located at 5 mm below fibular head at outer lateral posterior knee and puncture, an acupuncture needle was inserted perpendicularly to a depth of 7 mm; as for SP6, located at the tip of the inner ankle of the posterior limb, a needle was inserted upward 10 mm and perpendicularly to a depth of 5 mm; single-use disposable sterilized needles (0.3 mm × 25 mm) were used for the surgical procedures.

*EA Stimulation*. EA stimulation was conducted using a combination of two acupoints selected from “LU5, LI4” and “ST36, SP6.” Density wave was applied with frequency (2/15 Hz) and intensity (2 mA), which slightly shook the surrounding tissues around the acupoints. Duration for each EA stimulation was 20 minutes, and EA stimulation was conducted once daily at 3-day and 1-week time points; other time points were conducted only once.

#### 2.2.4. Evaluation of Neurological Deficit Scales

Neurological deficit scores were assessed based on the standard Longa's 5 grading scores method [21]. Multiple-blind approach was used to evaluate the score. Neurological deficit scores were defined as follows: (1) no neurological deficit, score 0; (2) failure to fully extend the contralateral forepaw, score 1; (3) spontaneous circling to the contralateral side, score 2; (4) falling to the opposite side, score 3; and (5) not spontaneously walking and loss of consciousness, score 4. Evaluation of neurological deficit scores was performed prior to tissue dissection.

#### 2.2.5. Morphological Evaluation by HE Staining

Slices were incubated with xylene, 100% ethanol for 5 minutes (twice), and 95%, 90%, and 80% ethanol each for 3 minutes, stained with hematoxylin for 10–15 minutes, and rinsed with water for 10 minutes. Following this, 1% hydrochloric acid alcohol was added for 1 second to visualize the blue color, which was followed by washing for 10 minutes. The brain tissue was stained with eosin for 5 minutes, dehydrated with 80%, 95%, and 100% ethanol for 1 minute (twice), and cleared with xylene for 2 × 5 minutes. Slices were mounted with neutral gum. The slices were photographed under a light microscope.

#### 2.2.6. Detection of Apoptosis by TUNEL Method

Routine deparaffinization and dehydration were performed for paraffin-embedded hippocampus section. The sections were incubated with protein K at RT for 15 min, washed in phosphate buffered saline (PBS) for 3 times (5 min/per time), and incubated under RT for 10 min. After the sections were rinsed in PBS for 3 times (5 min/per time), terminal deoxynucleotidyl transferase (TdT) and digoxin-labeled deoxyuridine triphosphate (dUTP) were mixed at a ratio of 1 : 9 (volume) and incubated in a water bath at 37°C for 60 min. Humidity was maintained by adding a small amount of water in a wet box. The sections were washed in PBS for 3 times; 4′,6-diamidino-2-phenylindole dihydrochloride (DAPI) was used for staining dried section. The section was incubated for 10 min at RT in the dark and was rinsed in PBS thrice; finally, the sections were mounted with antifade fluorescence mounting medium and were observed under a light microscope. The numbers of apoptotic cells and total cells were counted in 4 fields of CA1 area, which were chosen from each section. Apoptosis index (AI) was calculated according to the following formula:

(1) $$\begin{array}{c} \text{AI}=\left(\;\frac{\text{the number of apoptotic cells}}{\text{total cells}}\;\right)\times 100\%. \end{array}$$

#### 2.2.7. Detection of Expression of p-ERK by Immunohistochemistry

Hippocampus paraffin sections were routinely deparaffinized and dehydrated. Ethylenediaminetetraacetic acid (EDTA) antigen retrieval solution was used to retrieve antigen. 3% H_2_O_2_ was used for elimination of endogenous peroxidase and incubated for 20 min in the dark. The sections were soaked in PBS for 5 min and were dried; primary antibody, which is p-ERK antibody, was added to cover the sections. The sections were incubated overnight and flatly placed in a wet box. Secondary antibody rabbit anti-goat HRP was added after washing with PBS thrice (5 min), and the section was incubated for 50 min. PBS washing was repeated and 3,3′-diaminobenzidine (DAB) was added; positive cells were stained as brown yellow. Staining was terminated by rinsing with tap water. HE staining was carried out for 3 min, and the sections were washed with tap water following staining. The sections were differentiated in 70% ethanol containing 1% hydrochloric acid for few seconds and were dyed blue by ammonia. The sections were rinsed in tap water and dehydrated and cleared in different concentrations of alcohol and xylene; finally, the sections were dried and mounted in neutral gum in tap water. Image pro-plus 6.0 imaging analysis system was used for analyzing the images. p-ERK-positive cells were measured using mean grey value: higher grey value corresponded to lower level of p-ERK and vice versa.

#### 2.2.8. Western Blot Analysis

Hippocampus tissues were homogenized in Radio Immunoprecipitation Assay (RIPA) lysis buffer and centrifuged at 12,000 ×g for 5 min followed by determination of protein concentration in supernatants. Protein lysates were separated by 10% SDS-PAGE gels and then electrophoretically transferred onto PVDF membranes. The membranes were blocked for 1 h with 5% nonfat dry milk and then probed with primary antibodies against p-ERK, ERK, and actin (at a dilution of 1 : 3,000) overnight at 4°C, followed by incubation with appropriate HRP-conjugated secondary antibody for 30 min. Blots were developed using enhanced chemiluminescence, and images were obtained and analyzed using the alphaEaseFC analyzer software.

#### 2.2.9. Statistical Analysis

Statistical Package for the Social Sciences (SPSS) 20.0 software was used for the analysis of data. Data was expressed as mean ± standard deviation, which indicated that the data has been tested for normal distribution. If data followed normal distribution and homogeneity of variance test, Student's *t*-test or ANOVA was performed; if data did not follow a normal distribution and homogeneity of variance test, rank-sum test was performed for measurement of data from multiple groups.

## 3. Results

### 3.1. Effect of EA on Neurological Deficit Scores in Rats

**Figure 1:**
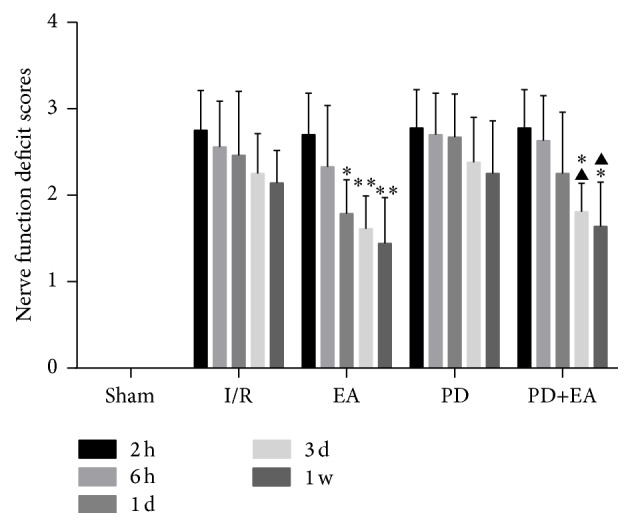
Bar graph showing the neurological deficit score in each group.

EA can reduce neurological deficit symptoms. After I/R injury, rats showed movement impairment and neurological deficiency. Neurological deficit scores at 1 day, 3 days, and 1 week in the EA group were lower than those in the I/R group (*p* < 0.05): neurological deficit scores in the EA and I/R groups at 3 days were 1.61 ± 0.38, 2.25 ± 0.46; 1.44 ± 0.53, 2.14 ± 0.38 at 1 week, respectively (*p* < 0.01). The scores were significantly improved in the PD+EA group at 3 days and 1 week when compared with those in the PD group (*p* < 0.05) (Figure 1).

### 3.2. Effect of EA on Morphological Change in Rats

**Figure 2:**
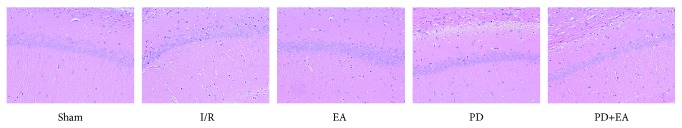
Morphological observation of brain tissue in each group under a light microscope (×200).

EA can reduce cerebral I/R injury in rats. Using HE staining method, pathological changes in the CA1 hippocampus in different groups were observed as follows: arrangement of pyramidal cells was tight and orderly, morphology of cells was normal, framework showed integrity with light staining, and nucleolus was clear in the hippocampus in the sham group (Figure 2). In the I/R group, the arrangement of cells in the hippocampal CA1 region was disordered and sparse, there was loss of nerve cells, and deformation was visible with nuclear pyknosis, unclear nucleolus, and dark staining. The morphological change was similar in both EA+PD and I/R groups. Compared with the I/R group, the arrangement of cells was less disordered and clear nucleolus, edema, and loss and deformation of the nerve cells were alleviated in the hippocampal CA1 region of EA group. However, nerve cell shrinkage and necrosis were severe in the PD group as compared to those in I/R group.

### 3.3. Effect of EA on Apoptotic Index in Rats

**Figure 3:**
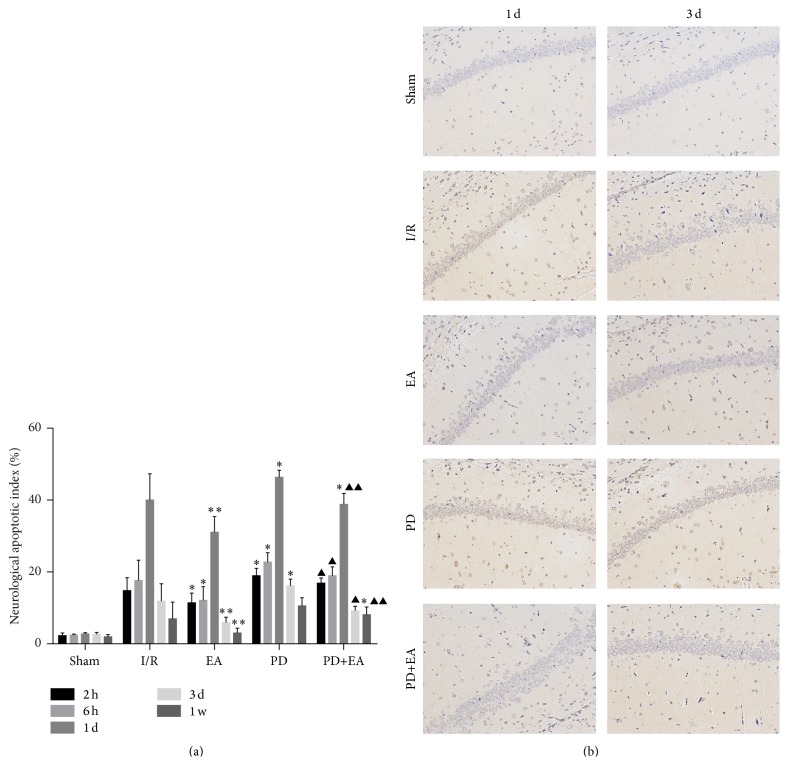
(a) Bar graph showing the apoptotic index of nerve cells. At the end of the experiment, cerebral tissues from each group (*n* = 8) were processed for TUNEL assay. Nuclei of all cells were visualized by DAPI staining, and apoptotic cells stained brown were detected by microscope. Apoptotic cells were counted at 4 arbitrarily selected microscopic fields at a magnification of ×200. Apoptotic rate was expressed as the ratio of brown-stained cells to the DAPI-stained total cells. Data is expressed as mean ± SE (error bars). At the same time point, versus I/R group, ^*∗*^ *p* < 0.05, ^*∗∗*^ *p* < 0.01; versus PD group, ^▲^ *p* < 0.05, ^▲▲^ *p* < 0.01. (b) Apoptosis of neural cells observed under a light microscope (TUNEL, ×200).

EA can decrease AI in rats subjected to cerebral I/R injury. Using TUNEL staining method, the trend of apoptosis at different time points in different groups was observed as follows: the number of apoptotic cells was increased 2 h after I/R injury; positive staining continuously increased and reached peak, decreased 1 day after I/R injury, and gradually became stable at 3 days after I/R injury (Figure 3(a)). When comparing AI at the same time point among different groups, AI in the EA group was significantly lower than that in the I/R group (*p* < 0.05). AI in the EA and I/R groups at 1 day was 31.06 ± 4.38% and 40.03 ± 7.28, respectively. AI in the EA and I/R groups at 3 days was 5.92 ± 1.51% and 11.89 ± 4.81%, respectively (*p* < 0.01). The rate of AI in the PD+EA group was lower than that in the PD group; the difference was evident at 1 day and 3 days (*p* < 0.01, Figure 3(a)).

Apoptosis in the CA1 hippocampus was observed in different groups at 1 day and 3 days (Figure 3(b)). A few apoptotic cells with positive stained nucleus were observed in the CA1 hippocampus in the sham group; a large number of positive apoptotic cells with positive stained nucleus were observed in the I/R group, and shrinkage of nucleus or chromatin margination was observed. AI was significantly increased in the I/R group as compared to sham group but relatively reduced when compared with PD group. The number of apoptotic cells in the hippocampal CA1 area in the EA and PD+EA groups was fewer than that in the I/R group.

### 3.4. Effect of EA on p-ERK Level in the Rats with I/R

**Figure 4:**
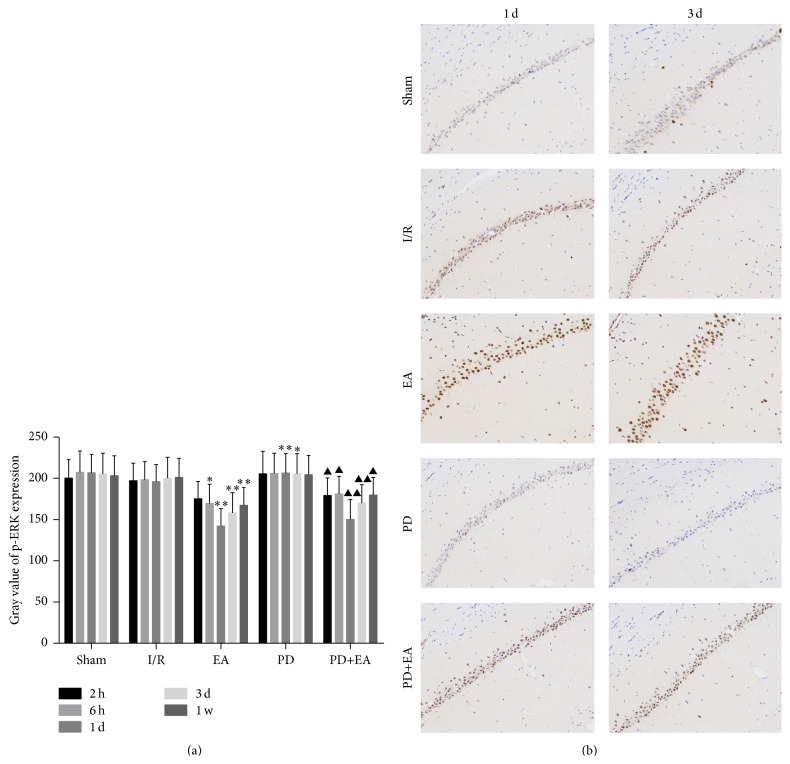
(a) Bar graph showing gray value of p-ERK level in the CA1 of the hippocampus. Data is representative of 8 individual rats in each group. Data is expressed as mean ± SE (error bars). At the same time point, versus I/R group, ^*∗*^ *p* < 0.05, ^*∗∗*^ *p* < 0.01; versus PD group, ^▲^ *p* < 0.05, ^▲▲^ *p* < 0.01. (b) Expression of p-ERK in the CA1 of the ipsilateral hippocampus (×200).

EA can upregulate the expression of p-ERK signaling pathway. p-ERK expression was detected using immunohistochemistry.  Figure 4(a) shows trend of p-ERK level in each group after I/R injury. p-ERK expression was upregulated 2 h after I/R injury in the EA group when compared with that in the I/R group (*p* < 0.05). Gray scales in the EA and I/R groups were 142.12 ± 21.34 and 196.21 ± 20.54 at 1 day, 158.31 ± 24.32 and 200.35 ± 25.21 at 3 days, and 167.42 ± 21.33 and 201.12 ± 23.23 at 1 week, respectively. There was a statistical difference between the two groups at these time points examined (*p* < 0.01). Moreover, p-ERK level was upregulated in the EA+PD group when compared with the PD group, and the upregulation was evident at 1 day and 3 days (Figure 4(a)). Occasionally, few p-ERK stained neurons were observed in the sham and I/R groups, while a large number of p-ERK stained neurons were observed in the EA group at 1 day and 3 days (Figure 4(b)). p-ERK level in the EA+PD group was lower than that in the EA group.

### 3.5. Effect of EA on p-ERK Protein Expression by Western Blot Analysis

**Figure 5:**
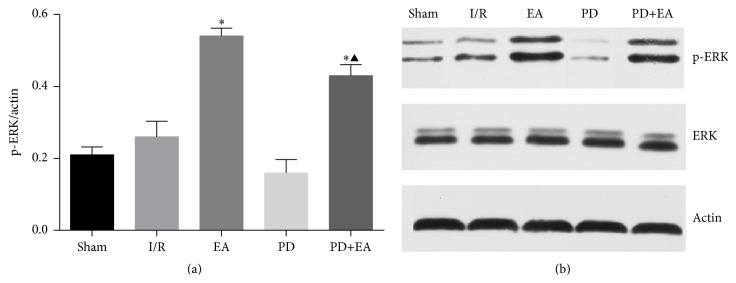
(a) The bar chart of p-ERK/actin in each of the five groups at 3-day time point (*n* = 8). Versus I/R group, ^*∗*^ *p* < 0.05. (b) The expressions of p-ERK and ERK were assessed by western blotting.

To further investigate the influence of electroacupuncture on the expression patterns of p-ERK, western blotting analysis was used to compare target protein levels at the 3-day time point of five groups. The western blotting data (Figure 5) verified the immunohistochemical findings (Figure 4) in that the protein expression levels of p-ERK were significantly higher in EA group and EA+PD group than in the I/R group (*p* < 0.05). p-ERK proteins were expressed at similar levels in sham and PD groups, while the lowest protein expression levels were found in PD group (Figure 5).

## 4. Discussion

EA of a combination of acupoints of “LU5,” “LI4,” “ST36,” and “SP6” significantly improves neurological deficiency symptoms, when compared with I/R group. Neurological deficit is an important index to evaluate cerebral I/R injury. Experimental studies have demonstrated that EA improved symptoms caused by I/R injury and promoted neurological functional recovery. Apoptosis in the neural cells contributes to cerebral I/R injury [23, 24]. In the present study, we observed apoptosis in the hippocampus in rats with cerebral I/R injury. Apoptosis of neural cells was evident when comparison was made between the sham group and other different surgery groups, which indicated that cerebral I/R injury could lead to delayed neuronal apoptosis. Moreover, apoptosis peaked at 1 day in all groups (except the sham group), reduced gradually, and became stable, which suggested that brain cells subjected to cerebral I/R injury have the capability of self-recovering for neurological function; however, there is certain limitation. A reduction in cellular apoptosis in the hippocampal CA1 area was observed in each of the EA subgroups, and there was a significant difference when compared with that of the I/R subgroup. This finding indicated that EA can inhibit cellular apoptosis induced by cerebral ischemia-reperfusion. Under a light microscope, we observed that cellular structures in the hippocampal CA1 area of EA group were relatively intact and well organized, whereas shrinkage and necrosis for a large amount of nerve cells were observed in the I/R group, which further demonstrated that EA had antiapoptotic effect on nerve cells and alleviated brain tissue damage. Relative research also showed that acupuncture was beneficial for antiapoptosis of nerve cells in the hippocampus and promoted cell recovery from cerebral I/R injury [25, 26]. EA exerts antiapoptotic effect through death receptor 5 (DR5) and by promoting the expression of antiapoptotic Bcl-2 and Bcl-xl in the mitochondria [27]. Additionally, EA also reduces neurotoxicity and exerts neuroprotective effect by inhibiting TNF-*α*/TRADD/FADD/cleaved caspase-8/cleaved caspase-3 apoptosis pathway [28]. Both previous studies and the present study demonstrated that EA inhibits apoptosis induced by cerebral I/R injury.

To study the mechanisms underlying antiapoptotic effect and neurological functional recovery of EA, we examined the expression of ERK signal pathway and chose CA1 area in the hippocampus as observation site. Immunohistochemistry was used to observe ERK expression. We observed that EA significantly increased p-ERK expression at 6 h, 1 day, 3 days, and 1 week in different subgroups when compared with the I/R group. And the western blotting data further verified the immunohistochemical findings, in that the protein expression levels of p-ERK were significantly higher in EA group than in the I/R group. In EA subgroups, with high expression of p-ERK, AI was relatively lower than that in the I/R group. It was assumed that antiapoptotic effect might be mediated through the upregulation of ERK signal pathway. Xie et al. [20] studied the effect of EA of “Quchi and Zusanli” on apoptosis induced by cerebral I/R injury and found that the mechanism of its antiapoptotic effect was mediated via upregulation of ERK, which was consistent with our study results. ERK signal pathway is a member of MAPK and exhibits neuroprotective effects [29]. Activation of ERK signal pathway has antiapoptotic effect and reduces brain injury after stroke [30]. Neuroprotective effect due to upregulation of ERK expression is mainly mediated by increasing antiapoptotic protein Bcl-2 or blocking the proapoptotic protein Bad to inhibit apoptosis [31, 32]. Additionally, antiapoptotic effects of neuroprotective growth factors, such as estrogen, on pretreatment of ischemia and lowering body temperature are associated with the upregulation of ERK expression [33]. Nevertheless, it has been shown that activation of ERK could also promote apoptosis induced by cerebral I/R injury [18], which mainly increases the damaging effect of inflammation and oxygen stress. Therefore, in the present study, in order to verify whether ERK upregulation has antiapoptotic effect, we established ERK inhibitor PD98059 and EA+PD98059 groups. We observed that PD98059 group not only inhibited ERK expression but also increased apoptosis, which indicated that upregulation of ERK expression signaling pathway has neuroprotective effect, whereas PD+EA group showed increased ERK expression when compared with the PD group and reduced cell apoptosis, which further confirmed that EA is associated with antiapoptotic activity mediated by the upregulation of ERK signaling pathway in rats with cerebral I/R injury.

Considering the design of the time points, we chose 1.5 h after I/R injury for EA intervention and observed after-effect of EA intervention at different time point subgroups on apoptosis. Research has shown that intervention within 1–4 h in acute phase of I/R injury could reduce the severity, reduces the number of dying neurons, alleviates apoptotic speed rate, and decreases expansion of infarction area [34–37]. Therefore, we chose 1.5 h after I/R for EA intervention and observed that apoptosis was reduced at different time points, and p-ERK expression was evident at 1 day and 3 days in the EA group, which gradually became stable. These findings verified that EA intervention reduced morbidity and mortality in acute phase I/R injury [38]. It has been shown that cellular apoptosis can occur within 30 min of reperfusion after 2 h of MCAO and peaked at 1 day or 2 days after surgery [39], whereas, in the present study, we observed that cellular apoptosis peaked within 1 day, decreased gradually, and became stable. It was opined that self-neural repair activating p-ERK expression when combined with active EA intervention could have synergistic effect in promoting neural repair. It has been suggested that ultra-early EA intervention is also associated with certain after-effects, 1 day time after EA intervention has better antiapoptosis of nerve cells. On the other hand, improvement of cellular apoptosis is evident at 3 days with once-daily EA intervention. It is assumed that better outcomes can only be achieved by continuous EA treatment for at least 3 days on the basis of ultra-early intervention in rats with cerebral I/R injury. Three days might become the best continuous chance for improvement of apoptosis of nerve cells, as the time allows for optimum treatment and reduces cell apoptosis.

## 5. Conclusion

EA of a combination of acupoints of “LU5,” “LI4,” “ST36,” and “SP6” can significantly improve neurological deficit and morphology of brain tissue and reduce apoptosis index of hippocampus. “Acupuncture stimulation-initiation of regulation of ERK signaling pathway-inhibition of cell apoptosis” might become one of key mechanisms of acupuncture treatment for cerebral I/R. Moreover, 1 day after cerebral I/R could become a better EA after-effect time point for antiapoptosis of neural cells, and 3 days after I/R is the best continuous EA treatment time for antiapoptosis.
